# Supplementary figures and images for: Comparative analysis of flavonoids, polyphenols and volatiles in roots, stems and leaves of five mangroves
Source: PeerJ. 2023 Jun 22;11:e15529. doi: 10.7717/peerj.15529 (PMC10290835; doi:10.7717/peerj.15529)

(a)

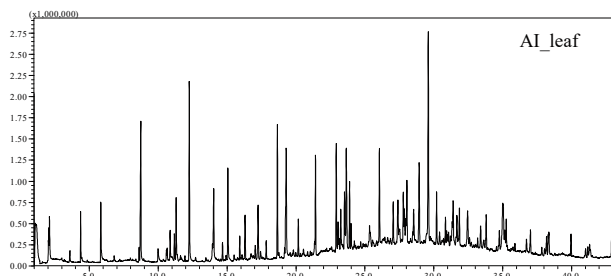

(b)

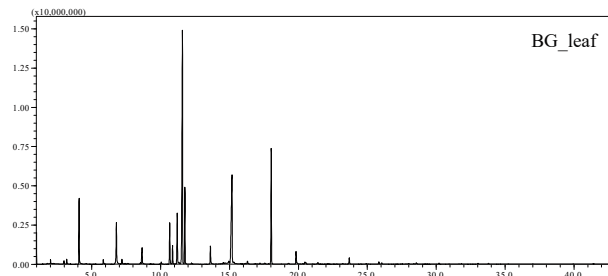

(c)

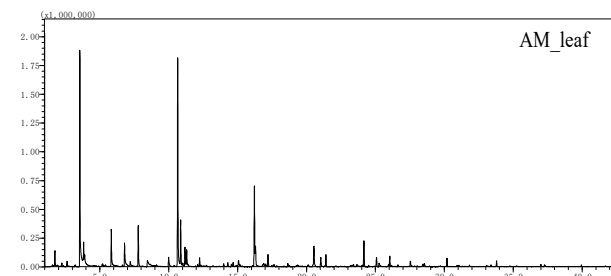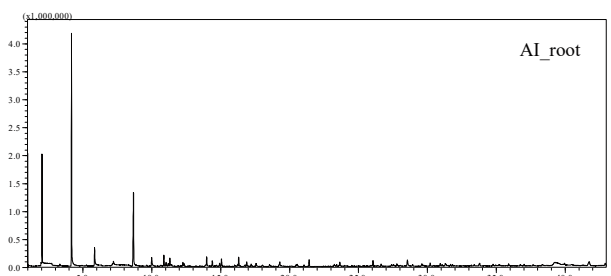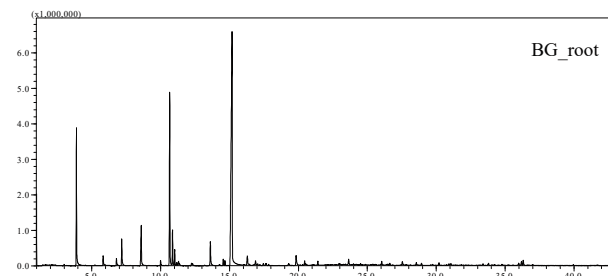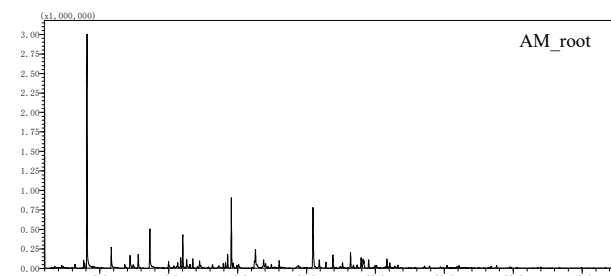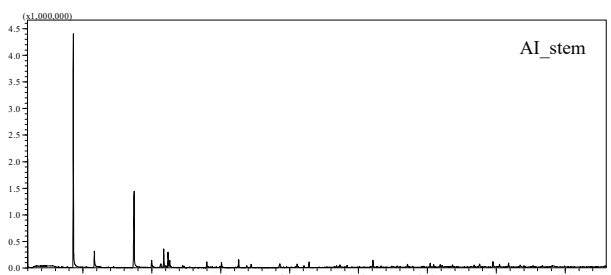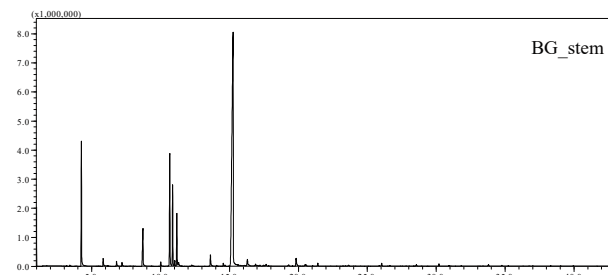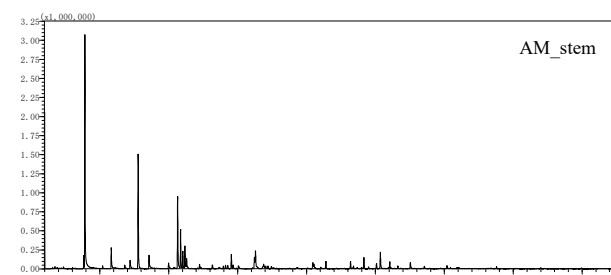

Supplement: Supplemental Information 1 — (a):A. ilicifolius (a), (b):K. candel, (c): A. marina. [file peerj-11-15529-s001.pdf]

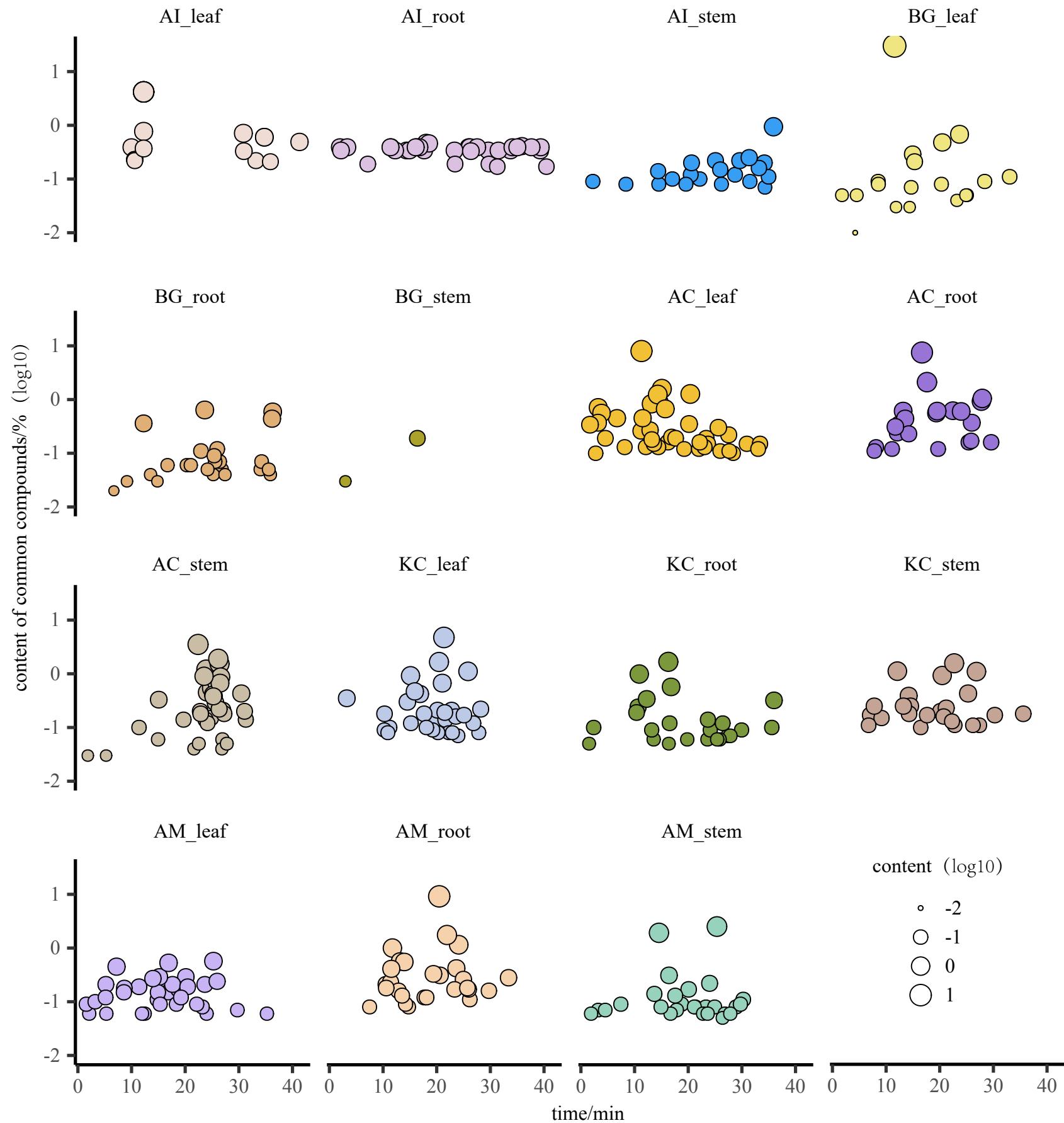

Supplement: Supplemental Information 2 — The horizontal axis indicates the retention time. The size of the circles indicate the relative content treated by log10 transformation. [file peerj-11-15529-s002.pdf]

- *A.corniculatum*
- *A.ilicifolius*
- *A.marina*
- *B.gymnorrhiza*
- *K.candel*

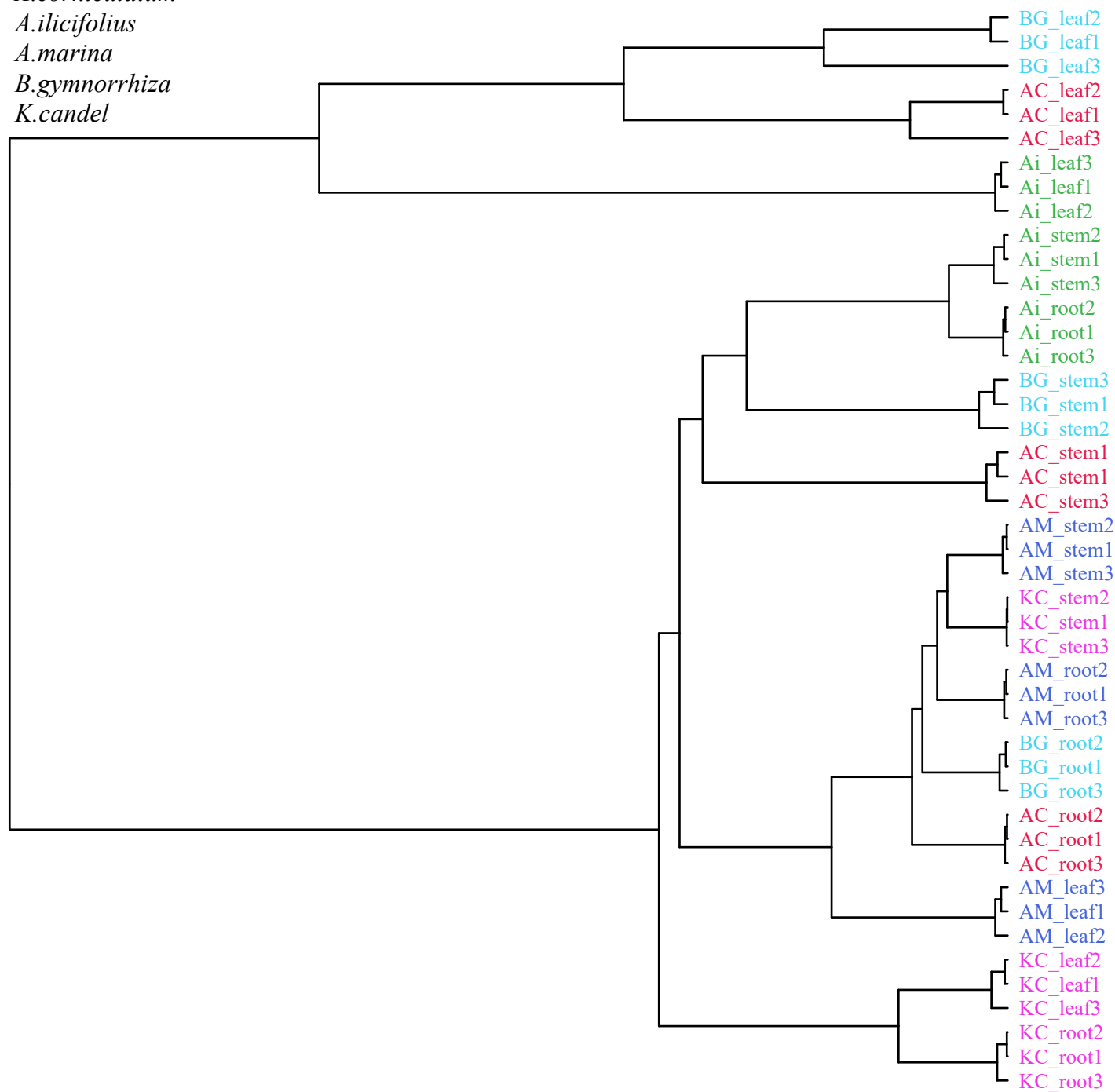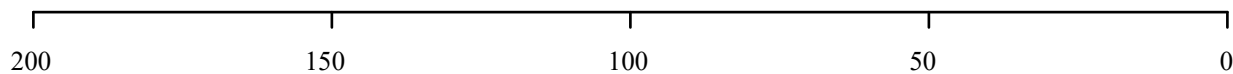

Supplement: Supplemental Information 3 — The numbers behind the texts on the right side of the figure indicate plant repeat numbers. Distance measure was used Euclidean distance. Ward’s method was applied in hierarchical cluster analysis. The clustering tree was made of two big classes, one of which was mainly composed of B.gymnorrhiza, A.corniculatum and A.ilicifolius leaves. Another big class was composed of three subclasses. Subclass 1: the stems and roots of A.ilicifolius had initially constituted a tiny subclass. then merged the stems of B.gymnorrhiza and A.cor niculatum to produce subclass 1; Subclass 2: stems of A.marina and K.candel created a little class, further united with the roots of K.candel, A.marina, A.corniculatum and B.gymnorrhiza, and the n combined with the leaves of A.marina; Subclass 3 was composed of the stems and leaves of K.candel. [file peerj-11-15529-s003.pdf]

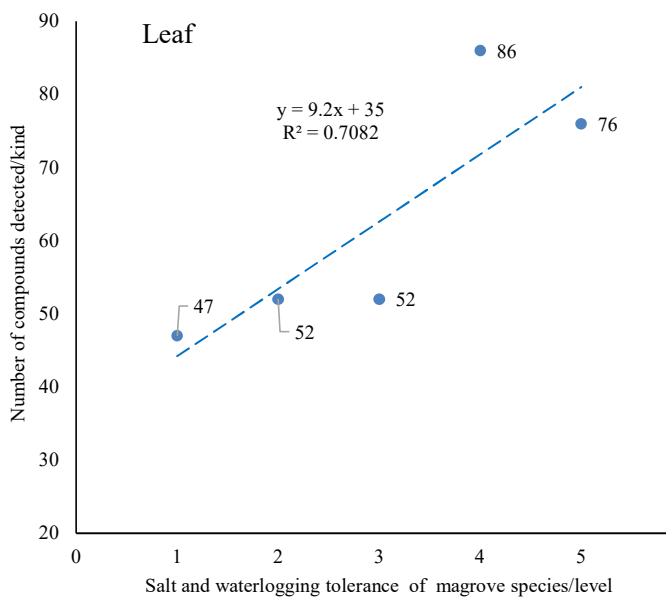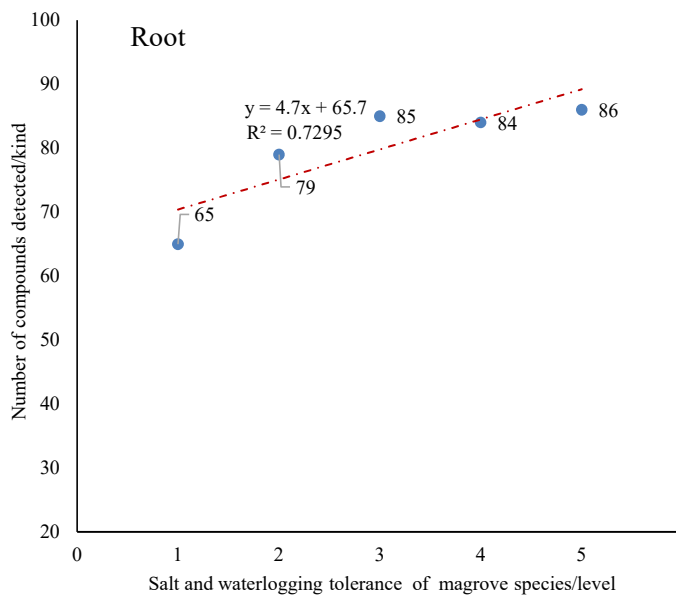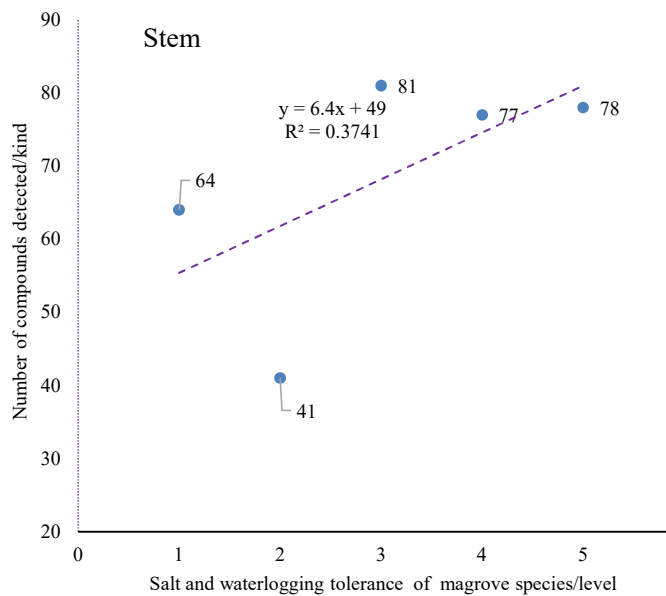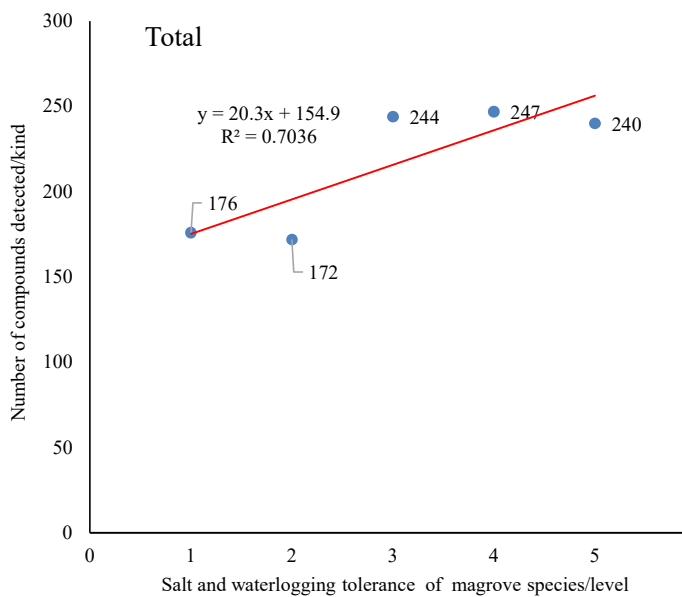

Supplement: Supplemental Information 4 — The vertical coordinate is the number of compounds in different parts/whole plants and the horizontal coordinate is mangrove tolerance levels of salt and waterlogging from weak to strong on a scale of 1–5 level. In each subplot, the equation and line represent the linear regression equation and trend line for the two variables. [file peerj-11-15529-s004.pdf]

## MONOTERPENOID BIOSYNTHESIS

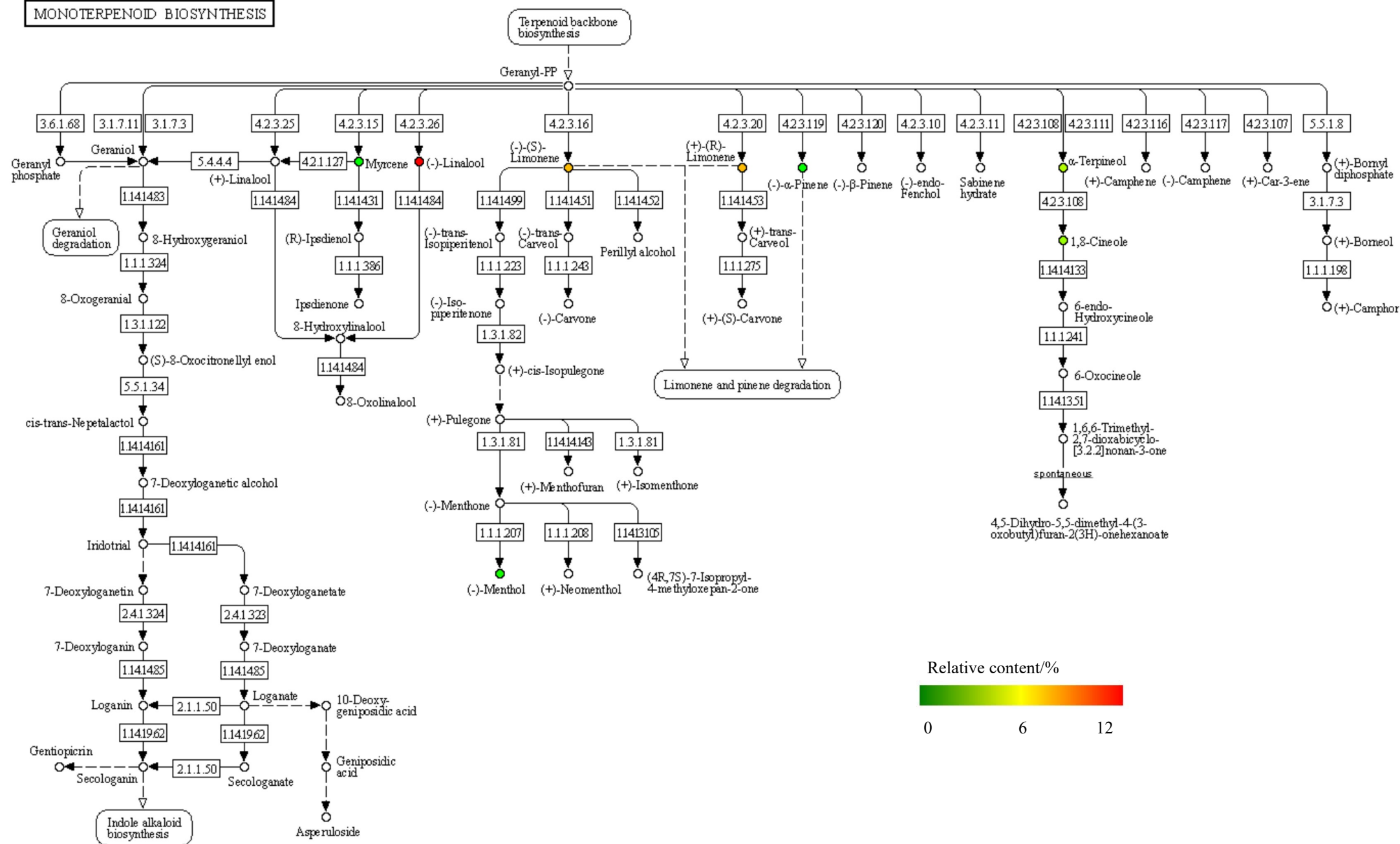

Supplement: Supplemental Information 5 — The colored dots in the metabolic pathway diagram are the detected metabolites. [file peerj-11-15529-s005.pdf]

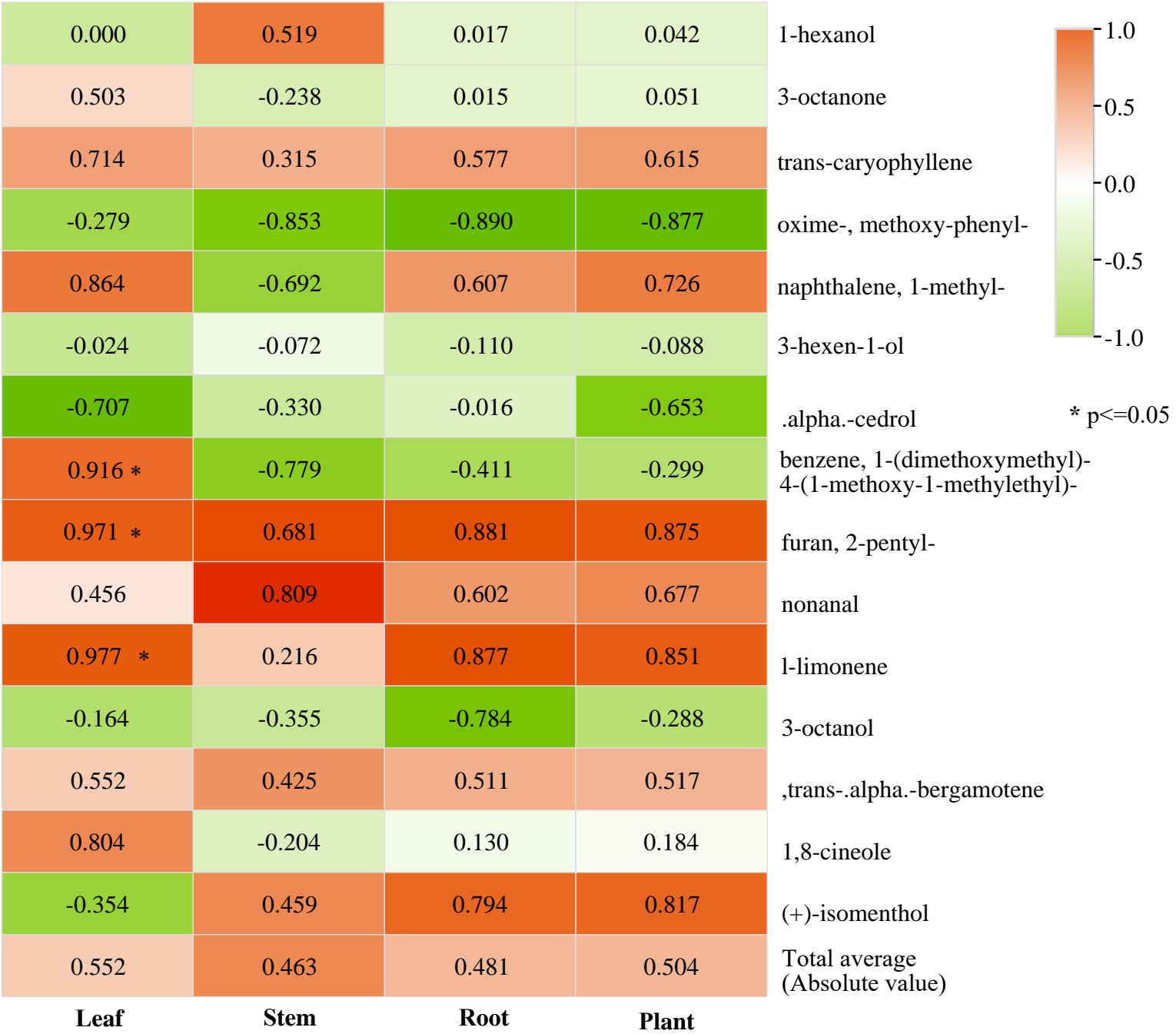

Supplement: Supplemental Information 6 — The differential compounds were found among mangrove species in Fig. 8. [file peerj-11-15529-s006.pdf]
