# Supplementary material for: Comparative analysis of flavonoids, polyphenols and volatiles in roots, stems and leaves of five mangroves
Source: PeerJ. 2023 Jun 22;11:e15529. doi: 10.7717/peerj.15529 (PMC10290835; doi:10.7717/peerj.15529)
Supplement: Supplemental Information 7 [file peerj-11-15529-s007.docx]

| **NO.** | **Compound name** | **Pubchem CID** | **KEEG ID** | **Total content/%** | **Classes** |
| --- | --- | --- | --- | --- | --- |
| 1 | (-)-.alpha.-Helmiscapene | 12309809 |  | 0.11 | Alkenes |
| 2 | (-)-endo-2,6-dimethyl-6-(4-methyl-3-pentenyl)bicyclo[3.1.1]hept-2-ene | 86608 |  | 0.07 | Alkenes |
| 3 | (+)-(1R,5R)-2(10)-PINEN-4-ONE | 65724 |  | 0.47 | Ketones |
| 4 | (+)-.BETA.-COSTOL | 12304104 |  | 0.17 | others |
| 5 | (+)-.BETA.-FUNEBRENE | 16213223 |  | 0.63 | Alkenes |
| 6 | (+)-2,6-dimethyl-6-(4-methyl-3-pentenyl)bicyclo[3.1.1]hep-2-ene | 6430742 |  | 0.09 | Alkenes |
| 7 | (+)-Aromadendrene | 91354 |  | 0.26 | Alkenes |
| 8 | (+)-CYCLOISOSATIVENE | 519960 |  | 3.52 | Ketones |
| 9 | (+)-Isomenthol | 6432468 |  | 8.81 | Alcohols |
| 10 | (+)-M-MENTHA-1,8-DIENE | 22311 | C06078 | 0.09 | others |
| 11 | (10 R)-Ethylisoxazoline - adduct from (5E)-Cholecalciferol | 23268453 |  | 0.21 | Alkaloids |
| 12 | (1R,2S,4R,6S)-4-Isopropenyl-1-methylbicyclo[4.1.0]heptan-2-ol | 101976768 |  | 0.22 | Alcohols |
| 13 | (1S,2E,6E,10R)-3,7,11,11-Tetramethylbicyclo[8.1.0]undeca-2,6-diene | 13894533 |  | 0.87 | Alkenes |
| 14 | (2,6,6-Trimethylcyclohex-1-enylmethanesulfonyl)benzene | 586248 |  | 0.38 | Sulfurs |
| 15 | (2RS,5RS)-2-ETHYL-1,6-DIOXASPIRO(4.4)NONANE | 581383 |  | 1.16 | Alkanes |
| 16 | (3aS,8aS)-6,8a-Dimethyl-3-(propan-2-ylidene)-1,2,3,3a,4,5,8,8a-octahydroazulene | 641722 |  | 1.9 | Alkenes |
| 17 | (3E)-2,6-DIMETHYL-3,7-OCTADIENE-2,6-DIOL # | 5352451 |  | 0.08 | Alcohols |
| 18 | (cis)-2-nonadecene | 5352531 |  | 0.44 | Alkenes |
| 19 | (E)-.beta.-Famesene |  |  | 2.4 | Alkenes |
| 20 | (E)-1-Methyl-4-(6-methylhept-5-en-2-ylidene)cyclohex-1-ene | 5352437 | C19748 | 0.21 | Alkenes |
| 21 | (E)-7-methyl-1,6-dioxaspiro[4.5]decane | 11116296 |  | 0.76 | Alkanes |
| 22 | (E)-Labda-8(17),12-diene-15,16-dial | 9904510 |  | 0.68 | others |
| 23 | (E,E)-1,3,5-Undecatriene | 5367412 |  | 0.18 | Alkenes |
| 24 | (E,E)-1,8-Dibromocyclotetradeca-1,8-diene | 11792107 |  | 0.11 | Alkenes |
| 25 | (R,S)-(1/,5u,7u)-7-(Hydroxymethyl)-7-methylbicyclo[3.3.1]nonan-2-one | 130027130 |  | 0.08 | Ketones |
| 26 | (S)-(E)-4-Phenyl-2-pentene | 12697652 |  | 0.12 | Alkenes |
| 27 | (S,1Z,6Z)-8-Isopropyl-1-methyl-5-methylenecyclodeca-1,6-diene | 91723653 |  | 1.35 | Alkenes |
| 28 | (Z)-(Z)-Hex-3-en-1-yl 2-methylbut-2-enoate | 16220109 |  | 2.4 | Esters |
| 29 | (Z)-.beta.-Farnesene | 5317319 |  | 0.26 | Alkenes |
| 30 | (Z)-3-hexenyl pentenoate | 129847996 |  | 0.57 | Alkenes |
| 31 | (Z)-4-Decen-1-ol | 5362798 |  | 0.12 | Alkenes |
| 32 | (Z)-CIS-.ALPHA.-BERGAMOTENE | 6428986 |  | 0.56 | Terpenoids |
| 33 | (Z)-Hex-3-enyl (E)-2-methylbut-2-enoate | 15461361 |  | 0.08 | Alkenes |
| 34 | ,TRANS-.ALPHA.-BERGAMOTENE | 6429302 |  | 12.15 | Alkenes |
| 35 | .alpha.-Amorphene | 101708 |  | 2.15 | Aldehydes |
| 36 | .alpha.-Cedrol | 6432709 | C09631 | 5.26 | Alkanes |
| 37 | .alpha.-Copaene | 12303902 | C09639 | 2.55 | Alkenes |
| 38 | .alpha.-Gurjunene | 521243 |  | 0.64 | Alkenes |
| 39 | .alpha.-Humulene | 5281520 | C09684 | 2.74 | Alkenes |
| 40 | .alpha.-Muurolene | 101708 | C20272 | 1.57 | Terpenoids |
| 41 | .alpha.-Pinene | 6654 | C06306 | 5.81 | Alkenes |
| 42 | .ALPHA.-PINENE, (-)- | 440968 | C06308 | 0.15 | Terpenoids |
| 43 | .ALPHA.-TERPINOLENE | 11463 | C06075 | 2.13 | Terpenoids |
| 44 | .alpha.-ylangene | 20055075 |  | 0.2 | Terpenoids |
| 45 | .beta. bisabolene | 68128 | C16775 | 1.53 | Alkenes |
| 46 | .beta.-Bisabolene | 10104370 | C16775 | 0.57 | Alkenes |
| 47 | .beta.-cadinene | 10657 | C09625 | 0.04 | Terpenoids |
| 48 | .beta.-Citronellol | 8842 |  | 0.46 | Alcohols |
| 49 | .beta.-Farnesene | 5281517 | C09666 | 0.48 | Alkenes |
| 50 | .beta.-Myrcene | 31253 | C06074 | 0.05 | Alkenes |
| 51 | .beta.-ylangene | 519779 |  | 0.44 | Alkenes |
| 52 | .delta.-(4)-dodecanol |  |  | 0.11 | Alcohols |
| 53 | .DELTA.3-Carene | 442461 | C09839 | 0.25 | Alkenes |
| 54 | .delta.-Cadinene | 12306054 |  | 1.1 | Terpenoids |
| 55 | .gamma.-Cadinene | 6432404 | C19738 | 0.12 | Terpenoids |
| 56 | :1,2,3,4,4a,9,10,10a -octahydro - 1,1,4a,7 - tramethyl - phenanthrene |  |  | 0.71 | polycyclic arenes |
| 57 | 1,1,1,2,2,3,3,4,4,5,5,6,6-Tridecafluorohexadec-7-ene | 2776160 |  | 0.09 | Alkenes |
| 58 | 1,1,7,12-tetramethyl-1,2,3,4,9,10,11,12-octahydrophenanthrene |  |  | 0.32 | polycyclic arenes |
| 59 | 1,10-Decanediol | 37153 |  | 0.33 | Alcohols |
| 60 | 1,1-Difluorooctane | 43606 |  | 0.23 | Alkanes |
| 61 | 1,1-dimethoxy decane | 158052605 |  | 0.25 | Alkanes |
| 62 | 1,2,3,5-tetramethylcyclohexane (1r,2c,3c,5t) |  |  | 0.07 | Alkanes |
| 63 | 1,2-Benzenedicarboxylic acid, bis(2-ethylhexyl) ester | 8343 | C03690 | 0.17 | Esters |
| 64 | 1,2-Benzenedicarboxylic acid, bis(2-methylpropyl) ester | 6782 | C15205 | 3.37 | Esters |
| 65 | 1,2-Benzenedicarboxylic acid, dibutyl ester | 3026 | C14214 | 1.56 | Esters |
| 66 | 1,2-Benzenedicarboxylic acid, diethyl ester | 6781 | C14175 | 4.62 | Esters |
| 67 | 1,2-Cyclohexanedicarboxylic acid, 2-biphenyl undecyl ester | 91722372 |  | 0.05 | Alkanes |
| 68 | 1,2-ETHANEDIOL, MONOFORMATE | 69404 |  | 0.17 | Alcohols |
| 69 | 1,3,3-Trimethyl-6-(3-nitro-benzenesulfonyl)-6-aza-bicyclo[3.2.1]octane | 2926859 |  | 1.58 | Sulfurs |
| 70 | 1,3,5,7-Cyclooctatetraene | 637866 |  | 18.85 | Alkenes |
| 71 | 1,3,6-Octatriene, 3,7-dimethyl-, (E)- | 5281553 | C09873 | 0.16 | Alkenes |
| 72 | 1,3,6-Octatriene, 3,7-dimethyl-, (Z)- | 5320250 | C09873 | 0.03 | Alkenes |
| 73 | 1,3-Cyclohexadiene, 5-(1,1-dimethylethyl)- | 556263 |  | 0.09 | Alkenes |
| 74 | 1,4-Benzenediol, 2,5-bis(1,1-dimethylethyl)- | 2374 |  | 0.71 | Alcohols |
| 75 | 1,4-Methanonaphthalene, 1,4-dihydro- | 97391 |  | 1.05 | Alkenes |
| 76 | 1,4-Octadiene | 5364369 |  | 0.12 | Alkenes |
| 77 | 1,5-PENTANEDIOL, 3-METHYL- | 20524 |  | 0.07 | Alcohols |
| 78 | 1,5-Pentanediol, O,O'-di(propargyloxycarbonyl) ester | 91694662 |  | 0.09 | Alcohols |
| 79 | 1,6,10-Dodecatriene, 7,11-dimethyl-3-methylene- | 5281517 | C09666 | 0.7 | Alkenes |
| 80 | 1,6,10-DODECATRIENE, 7,11-DIMETHYL-3-METHYLENE-, (E)- | 5281517 | C09666 | 1.11 | Alkenes |
| 81 | 1,6-Dioxaspiro[4.4]nonane, 2-ethyl- | 581383 |  | 0.55 | Alkanes |
| 82 | 1,6-OCTADIEN-3-OL, 3,7-DIMETHYL- | 6549 | C03985 | 1.92 | Alcohols |
| 83 | 1,6-Octadien-3-ol, 3,7-dimethyl-, (.+-.)- | 12452972 |  | 0.25 | Alkenes |
| 84 | 1,7-Nonadiene, 4,8-dimethyl- | 534956 |  | 0.26 | Alkenes |
| 85 | 1,8-Cineole | 2758 | C09844 | 3.68 | Alkenes |
| 86 | 1,E-8,Z-10-Tetradecatriene | 5365581 |  | 0.93 | Alkenes |
| 87 | 10,12-Octadecadiynoic acid | 522487 |  | 0.05 | Alkenes |
| 88 | 10-BROMO-7-HYDROXY-11-IODOLAURENE | 23425301 |  | 0.06 | Alkenes |
| 89 | 10-Methyl-8-tetradecen-1-ol acetate | 5363228 |  | 0.14 | Alkenes |
| 90 | 10-Undecyn-1-ol | 76015 |  | 0.26 | Alkenes |
| 91 | 11,14-Octadecadiynoic acid, methyl ester | 539208 |  | 0.67 | Esters |
| 92 | 1-Acetyl-2-hydroxy-3-methoxy-9-methyl-8,9-dihydronaphtho[d]benzo[b]furan-11(10)H-one |  |  | 0.06 | others |
| 93 | 1-Butanol | 263 | C06142 | 0.1 | Alcohols |
| 94 | 1-Butanol, 2-methyl- | 8723 |  | 1.93 | Alcohols |
| 95 | 1-Butanol, 2-methyl-, (S)- | 2723602 |  | 0.58 | Alcohols |
| 96 | 1-Butanol, 2-methyl-, acetate | 12209 |  | 0.08 | Alcohols |
| 97 | 1-BUTANOL, 3-METHYL- | 31260 | C07328 | 0.56 | Alcohols |
| 98 | 1-Butanol, 3-methyl- (impure) |  | C07329 | 4.99 | Alcohols |
| 99 | 1-Butanol, 3-methyl-, acetate | 31276 | C12296 | 0.58 | Alcohols |
| 100 | 1-Butylpentyl trifluoromethanesulfonate |  |  | 0.08 | Esters |
| 101 | 1-chloro-1,1,2-trifluoro-2-tridecene |  |  | 0.1 | Alkenes |
| 102 | 1-Chloro-2,2,4-trimethylpentan-3-ol |  |  | 0.11 | Alcohols |
| 103 | 1-CYCLOPROPYL-1-FLUOROETHANE |  |  | 0.09 | Alkanes |
| 104 | 1-Decanol | 8174 | C01633 | 4.15 | Alcohols |
| 105 | 1-DODECENE | 8183 |  | 1.34 | Alkenes |
| 106 | 1-Eicosanol | 12404 |  | 0.09 | Alcohols |
| 107 | 1-Ethynyl-1-cycloheptanol | 137728 |  | 0.14 | Alcohols |
| 108 | 1H-3a,7-Methanoazulene, 2,3,4,7,8,8a-hexahydro-3,6,8,8-tetramethyl-, [3R-(3.alpha.,3a.beta.,7.beta.,8a.alpha.)]- | 521207 |  | 0.6 | Terpenoids |
| 109 | 1H-Benzocycloheptene, 2,4a,5,6,7,8-hexahydro-3,5,5,9-tetramethyl-, (R)- | 11586487 |  | 0.22 | Alkenes |
| 110 | 1-Heptanol | 8129 |  | 1.31 | Alcohols |
| 111 | 1-Heptanol, 3-methyl- | 517883 |  | 0.09 | Alcohols |
| 112 | 1-Hepten-4-ol | 19040 |  | 0.08 | Alcohols |
| 113 | 1-Hexadecene | 12395 |  | 0.12 | Alkenes |
| 114 | 1-Hexadecyne | 12396 |  | 0.17 | Alkenes |
| 115 | 1-HEXANOL | 8103 | C00854 | 36.4 | Alcohols |
| 116 | 1-Hexanol, 5-methyl- | 12331 |  | 0.08 | Alcohols |
| 117 | 1-Hexene, 2-methyl- | 22468 |  | 0.18 | Alkenes |
| 118 | 1-Hexene, 3,5,5-trimethyl- | 92984 |  | 0.89 | Alkenes |
| 119 | 1H-Indene, 1-ethylidene- | 5373026 |  | 2.21 | Aromatic hydrocarbons |
| 120 | 1H-Indene, 1-methylene- | 75581 |  | 2.84 | Aromatic hydrocarbons |
| 121 | 1H-Purin-6-amine, [(2-fluorophenyl)methyl]- |  |  | 0.49 | Nucleotides and derivatives |
| 122 | 1-Iodo-2-methylnonane | 537317 |  | 1.51 | Alkanes |
| 123 | 1-METHYL-2-PROPYNYL DIETHYLBORINATE # | 543036 |  | 0.1 | others |
| 124 | 1-Methyl-4-(2-propyl)cyclooctan-1-ol |  |  | 0.11 | Alcohols |
| 125 | 1-METHYL-CIS-2-(N,N-DIMETHYLAMINOMETHYL-D2)ISOPROPYLIDENECYCLOPENTANE |  |  | 0.03 | Alkanes |
| 126 | 1-Methylcyclohexanol, trifluoroacetate | 6432711 |  | 0.71 | Alcohols |
| 127 | 1-Nonanol | 8914 | C14696 | 0.69 | Alcohols |
| 128 | 1-Octadecanol | 8221 | D01924 | 0.1 | Alcohols |
| 129 | 1-OCTANOL | 957 | C00756 | 7.49 | Alcohols |
| 130 | 1-OCTANOL, 2-BUTYL- | 19800 |  | 0.05 | Alcohols |
| 131 | 1-Octen-3-ol | 18827 | C14272 | 61.26 | Alkenes |
| 132 | 1-Octen-3-one | 61346 |  | 0.59 | Ketones |
| 133 | 1-octen-3-yl acetate | 17121 |  | 0.29 | Alkenes |
| 134 | 1-Pentanol | 6276 | C16834 | 0.62 | Alcohols |
| 135 | 1-Pentanol, 4-methyl-2-propyl- | 103862 |  | 0.07 | Alcohols |
| 136 | 1-Pentanol, 5-cyclopropylidene- | 551450 |  | 0.57 | Alcohols |
| 137 | 1-Penten-3-ol | 12020 |  | 1.12 | Alkenes |
| 138 | 1-Penten-3-one, 1-(2,6,6-trimethyl-1-cyclohexen-1-yl)- | 5375218 |  | 0.07 | Ketones |
| 139 | 1-phenyl-3-methylpenta-1,2,4-triene | 593639 |  | 0.15 | Alkenes |
| 140 | 1-Propanol, 2,2-dimethyl- | 6404 |  | 0.13 | Alkenes |
| 141 | 1-Propene, 2-fluoro- | 70899 |  | 0.06 | Alkenes |
| 142 | 1-Tetradecanol | 8209 | D05097 | 2.12 | Alcohols |
| 143 | 1-Tridecene | 17095 |  | 0.04 | Alkenes |
| 144 | 1-Undecanol | 8184 |  | 0.39 | Alcohols |
| 145 | 2 OCTENAL | 5283324 |  | 0.61 | Alkenes |
| 146 | 2(1H)-Naphthalenone, 3,4,4a,5,6,7-hexahydro-1,1,4a-trimethyl- | 535380 |  | 0.2 | Ketones |
| 147 | 2-(1-hydroxy ethyl)-5-methyl-5-vinyl-tetrahydrofuran |  |  | 0.1 | Others |
| 148 | 2(3H)-Furanone, dihydro- | 7302 | C01770 | 0.18 | Others |
| 149 | 2,2,6,6-Tetramethylcyclohexan-1-one | 136933 |  | 0.06 | Ketones |
| 150 | 2,2-Dimethylpropanoic acid, 3-methylbut-2-enyl ester | 531134 |  | 0.67 | Alkenes |
| 151 | 2,3,5,8,10,11-HEXAMETHYLENEDODECANE |  |  | 0.08 | Alkanes |
| 152 | 2,4 PENTADIENAL | 5986428 |  | 0.03 | Alkenals |
| 153 | 2,4(1H,3H)-Pyrimidinedione, 5-nitro- | 69135 |  | 0.06 | Nucleotides and derivatives |
| 154 | 2,4-Dimethyl 1,4-pentadiene | 138137 |  | 0.12 | Alkenes |
| 155 | 2,4-O-Benzylidene d-glucitol | 247001 |  | 0.19 | Alcohols |
| 156 | 2,5,5,8a-Tetramethyl-3,4,4a,5,6,8a-hexahydro-2H-chromene | 6432687 |  | 1.66 | Alkenes |
| 157 | 2,5,5-TRIMETHYL-CYCLOHEX-2-EN-1-ONE | 3827783 |  | 0.16 | Ketones |
| 158 | 2,5,5-Trimethylcyclohex-2-enone | 586631 |  | 0.23 | Ketones |
| 159 | 2,5-cyclohexadien-1-one, 2,6-bis(1,1-dimethylethyl)-4-hydroxy-4-methyl- | 146102 |  | 0.09 | Ketones |
| 160 | 2,5-Dihydroxybenzaldehyde, 2TMS derivative | 622536 |  | 3.46 | Aldehydes |
| 161 | 2,5-Dimethylbenzophenone | 256066 |  | 0.39 | Ketones |
| 162 | 2,6,10 - trimethyl - tridecane (WITHOUT stereochemistry) |  |  | 0.11 | Alkanes |
| 163 | 2,6-di-butyl-2,5-cyclohexadiene-1,4-dione |  |  | 0.65 | Ketones |
| 164 | 2,6-DIHYDROXYBENZOIC ACID 3TMS |  |  | 0.15 | Acids |
| 165 | 2-[Ethoxymethoxy]-1,3-propanediol |  |  | 0.15 | Alcohols |
| 166 | 2-Acetyl-5-norbornene | 107366 |  | 0.05 | Alkenes |
| 167 | 2-Allyl-1,3-cyclohexanedione | 566005 |  | 0.14 | Ketones |
| 168 | 2-BUTEN-1-OL, 3-METHYL- | 11173 | C01390 | 0.08 | Alkenes |
| 169 | 2-Cyanomethyl-tetrahydrofuran | 4962721 |  | 0.17 | Others |
| 170 | 2-Cyclohexen-1-one, 3-methyl- | 14511 |  | 0.52 | Ketones |
| 171 | 2-Cyclohexen-1-one, 3-methyl-6-(1-methylethyl)- | 6987 |  | 0.4 | Ketones |
| 172 | 2-Decanone | 12741 |  | 0.18 | Ketones |
| 173 | 2-Decenal, (E)- | 5283345 |  | 0.61 | Alkenals |
| 174 | 2-Decyn-1-ol | 77763 |  | 0.36 | Alcohols |
| 175 | 2-Dodecenal, (E)- | 5283361 |  | 0.62 | Alkenals |
| 176 | 2-Ethyl-1-hexanol | 7720 | C02498 | 4.19 | Alcohols |
| 177 | 2-Hepten-1-ol, (E)- | 5318017 |  | 0.06 | Alcohols |
| 178 | 2-Heptenal, (E)- | 5283316 |  | 0.34 | Alkenals |
| 179 | 2-Heptenoic acid, ethyl ester, (E)- | 5358363 |  | 6.26 | Alkenes |
| 180 | 2-Heptyn-1-ol | 535111 |  | 0.31 | Alcohols |
| 181 | 2-Hexanone, 6-bromo- | 25061 |  | 0.09 | Ketones |
| 182 | 2-Hexen-1-ol, (E)- | 5318042 |  | 0.35 | Ketones |
| 183 | 2-Methoxy-1,3-dioxolane | 29744 |  | 0.3 | Others |
| 184 | 2-Methylamino-1-phenylethanol | 913 | C03711 | 0.06 | Alcohols |
| 185 | 2-Nonanone | 13187 |  | 3.03 | Ketones |
| 186 | 2-Nonen-1-ol, (Z)- | 5365027 |  | 0.07 | Alkenes |
| 187 | 2-Nonenal, (E)- | 5283335 |  | 1.11 | Alkenals |
| 188 | 2-Nonenal, (Z)- | 5354833 |  | 0.88 | Alkenals |
| 189 | 2-Octen-1-ol | 5318599 |  | 0.44 | Alkenes |
| 190 | 2-Octen-1-ol, (E)- | 5318599 |  | 0.04 | Alkenes |
| 191 | 2-Octenal, (E)- | 5283324 |  | 0.47 | Alkenals |
| 192 | 2-Oxonanone | 138568 |  | 0.22 | Ketones |
| 193 | 2-Pentadecanone | 61303 |  | 0.19 | Ketones |
| 194 | 2-Pentadecanone, 6,10,14-trimethyl- | 10408 |  | 0.2 | Ketones |
| 195 | 2-Penten-1-ol, (E)- | 5364920 |  | 0.19 | Alkenes |
| 196 | 2-Penten-1-ol, (Z)- | 5364919 |  | 1 | Alkenes |
| 197 | 2-Penten-1-ol, acetate, (Z)- | 5363400 |  | 0.09 | Alkenes |
| 198 | 2-Pentene, (Z)- | 5326160 |  | 0.34 | Alkenes |
| 199 | 2-Propenamide, 2-methyl-N-phenyl- | 74164 |  | 0.19 | Alkenes |
| 200 | 2-Propenoic acid, 3-phenyl-, ethyl ester | 637758 | C06359 | 0.57 | Alkenes |
| 201 | 2-Pyrazolin-5-ol, 5-tert-butyl-3-trifluoromethyl-1-(3-methylbenzoyl)- | 576778 |  | 0.12 | Alcohols |
| 202 | 2-tert-Butyltoluene | 33712 |  | 0.21 | monocyclic aromatics |
| 203 | 2-Tridecanone | 11622 |  | 0.12 | Ketones |
| 204 | 2-Undecanone | 8163 | C01875 | 3.08 | Ketones |
| 205 | 3-(2-Methyl-propenyl)-1H-indene | 593413 |  | 0.18 | Alkenes |
| 206 | 3-(Hex-1'-enyl)cyclopropane-2,2-dimethylcyclopropane-1-methanol |  |  | 0.08 | Alkenes |
| 207 | 3-(Hydroxyphenylmethyl)-2-methyl-3-buten-1-ol |  |  | 0.18 | Alkenes |
| 208 | 3,3'-Diisopropyl-1,1'-bis(bicyclo[1.1.1]pentane] |  |  | 0.06 | Alkanes |
| 209 | 3,4-DIHYDROXYMANDELIC ACID-TETRATMS |  |  | 0.67 | Acids |
| 210 | 3,4-Hexadienal, 2-butyl-2-ethyl-5-methyl- | 252498 |  | 0.16 | Aldehydes |
| 211 | 3-Acetyl-2-methyl-9b-(1-naphthyl)-3aH-benzo[e]cyclohexa[1,2-b]furan |  |  | 1.01 | polycyclic arenes |
| 212 | 3-BUTEN-2-OL, 2-METHYL- | 8257 |  | 0.09 | Alkenes |
| 213 | 3-Buten-2-one | 6570 | C20701 | 0.05 | Ketones |
| 214 | 3-Cyclohexen-1-ol, 4-methyl-1-(1-methylethyl)- | 11230 | C17073 | 4.44 | Terpenoids |
| 215 | 3-Cyclohexene-1-carboxaldehyde, 1,3,4-trimethyl- | 38621 |  | 0.09 | Alkenes |
| 216 | 3-Cyclohexene-1-methanol, .alpha.,.alpha.,4-trimethyl-, (S)- | 443162 | C11393 | 4.29 | Alkenes |
| 217 | 3-Dodecanol, 3,7,11-trimethyl- | 23701 |  | 0.2 | Alcohols |
| 218 | 3H-1,2-Dithiole | 525331 |  | 0.08 | Alcohols |
| 219 | 3-Heptanol, 3,6-dimethyl- | 15297 |  | 0.04 | Alcohols |
| 220 | 3-Hexen-1-ol | 5284503 | C08492 | 48.14 | Alkenes |
| 221 | 3-Hexen-1-ol, (E)- | 5284503 | C08492 | 0.22 | Alkenes |
| 222 | 3-Hexen-1-ol, (Z)- | 5281167 | C08492 | 1 | Alkenes |
| 223 | 3-Hexen-1-ol, 2,5-dimethyl-, formate,(Z)- | 5367771 |  | 0.67 | Alkenes |
| 224 | 3-HEXEN-1-OL, ACETATE, (Z)- | 5363388 | C19757 | 30.57 | Alkenes |
| 225 | 3-Hexen-1-ol, benzoate, (Z)- | 5367706 |  | 3.61 | Alkenes |
| 226 | 3-Hexen-1-ol, propanoate, (Z)- | 5365049 |  | 0.3 | Alkenes |
| 227 | 3-Hexenoic acid, ethyl ester, (Z)- | 5362623 |  | 0.45 | Alkenes |
| 228 | 3-Hydroxy-7,8-dihydro-.beta.-ionol | 519379 |  | 0.43 | Alcohols |
| 229 | 3-methyl butanol | 31260 | C07328 | 0.07 | Alcohols |
| 230 | 3-Methyl-4-(phenylthio)-2-prop-2-enyl-2,5-dihydrothiophene 1,1-dioxide | 6420230 |  | 0.6 | Alkenes |
| 231 | 3-Methylpent-2-ene-1,5-diol | 5362855 |  | 0.08 | Alcohols |
| 232 | 3-Octanol | 11527 | C17144 | 20.94 | Alcohols |
| 233 | 3-Octanone | 246728 | C17145 | 29.37 | Ketones |
| 234 | 3-Octyn-1-ol | 84694 |  | 0.12 | Alkenes |
| 235 | 3-PENTANONE | 7288 |  | 0.74 | Ketones |
| 236 | 3-TETRADECENE, (E)- | 5352802 |  | 4.34 | Alkenes |
| 237 | 3-Tetradecene, (Z)- | 5362709 |  | 1.27 | Alkenes |
| 238 | 3-Trifluoroacetoxypentadecane | 534406 |  | 0.2 | Alkanes |
| 239 | 4-(2',6',6'-TRIMETHYLCYCLOHEX-1'-EN-1'-YL)BUTAN-1-OL |  |  | 0.15 | Alcohols |
| 240 | 4-(2-Methoxypropan-2-yl)-1-methylcyclohex-1-ene | 85755 |  | 0.7 | Alkenes |
| 241 | 4-Amino-furazan-3-carboxylic acid (3-morpholin-4-yl-propyl)-amide | 567684 |  | 0.37 | Others |
| 242 | 4-DECENE, 7-METHYL-, (E)- | 5363240 |  | 0.34 | Alkenes |
| 243 | 4D-METHYLHEXANOIC ACID ETHYL ESTER |  |  | 0.15 | Esters |
| 244 | 4-Ethyl-5-methyl-1,2-diazine | 86255064 |  | 0.06 | others |
| 245 | 4-Fluoro-2-methoxyphenol | 2737368 |  | 0.08 | Phenols |
| 246 | 4-Tetradecene, (E)- | 5364426 |  | 2.86 | Alkenes |
| 247 | 5,5-Dimethyl-cyclohex-3-en-1-ol | 546038 |  | 0.07 | Alcohols |
| 248 | 5,7-Octadien-2-ol, 2,6-dimethyl- | 5368785 |  | 0.14 | Alcohols |
| 249 | 5,8-Dimethylenebicyclo[2.2.2]oct-2-ene | 562340 |  | 0.23 | Alkenes |
| 250 | 5-Azulenemethanol, 1,2,3,4,5,6,7,8-octahydro-.alpha.,.alpha.,3,8-tetramethyl- | 521245 |  | 0.12 | Alcohols |
| 251 | 5-Hepten-2-ol, 6-methyl- | 20745 | C07288 | 0.53 | Alkenes |
| 252 | 5-Isopropenyl-1,2-dimethylcyclohex-2-enol | 536558 |  | 0.21 | Alkenes |
| 253 | 6-[(Z)-1-Butenyl]-1,4-cycloheptadiene | 5367429 |  | 0.29 | Alkenes |
| 254 | 6-Butyl-1,4-cycloheptadiene | 556470 |  | 0.21 | Alkenes |
| 255 | 6-Methyl-3-phenethylsulfanyl-[1,2,4]triazin-5-ol | 135399067 |  | 0.33 | Sulfurs |
| 256 | 6-Methyl-hept-5-en-2-ol | 20745 | C07288 | 0.12 | Alcohols |
| 257 | 6-Nonen-1-ol, (E)- | 5362811 |  | 0.06 | Alkenes |
| 258 | 6-Tridecanone | 89156 |  | 2.51 | Ketones |
| 259 | 7,9-Di-tert-butyl-1-oxaspiro(4,5)deca-6,9-diene-2,8-dione | 545303 |  | 0.94 | others |
| 260 | 7-chloro-1,3-dihydro-5-phenyl-2h-1,4-dibenzodiazepin-2-one tms de |  |  | 0.14 | Ketones |
| 261 | 7-Octen-3-ol, 2,6-dimethyl- | 565253 |  | 0.09 | Alkenes |
| 262 | 7-Octen-4-ol | 40923 |  | 0.84 | Alkenes |
| 263 | 7-Oxabicyclo[4.1.0]heptane, 3-methyl- | 535184 |  | 0.56 | Alkanes |
| 264 | 9-OCTADECENE, (E)- | 5364599 |  | 0.09 | Alkenes |
| 265 | Acetic acid, 2-ethylhexyl ester | 7635 |  | 12.38 | Esters |
| 266 | Acetic acid, decyl ester | 8167 |  | 0.68 | Esters |
| 267 | Acetic acid, ethyl ester | 8857 | C00849 | 0.6 | Esters |
| 268 | Acetic acid, heptyl ester | 8159 |  | 0.44 | Esters |
| 269 | Acetic acid, hexyl ester | 8908 |  | 7.97 | Esters |
| 270 | Acetic acid, non-3-enyl ester, cis- | 71586960 |  | 9.17 | Alkenes |
| 271 | Acetic acid, nonyl ester | 8918 |  | 1.19 | Esters |
| 272 | ACETIC ACID, PENTYL ESTER | 12348 |  | 0.08 | Esters |
| 273 | Acetic acid, thiocyanato-, 1,7,7-trimethylbicyclo[2.2.1]hept-2-yl ester, exo- | 6431949 |  | 0.06 | Esters |
| 274 | Acetic acid, trifluoro-, 3,7-dimethyloctyl ester | 537083 |  | 0.71 | Esters |
| 275 | Acoradiene | 90351 |  | 0.69 | Aromatic hydrocarbons |
| 276 | Actaldehyde ethyl benzyl acetal |  |  | 0.06 | Alcohols |
| 277 | Alloaromadendrene | 91354 |  | 0.43 | alkenes |
| 278 | Andrographolide | 5318517 | C20214 | 1.91 | Esters |
| 279 | A-PHELLANDRENE | 7460 |  | 0.3 | Terpenoids |
| 280 | Azulene | 9231 | C13392 | 1.6 | Alkenes |
| 281 | Benzamide, N-[1,3-dioxo-2-(1-imidazolyl)-2-indanyl]- | 569626 |  | 0.3 | polycyclic arenes |
| 282 | BENZENE, (1,1-DIMETHYLETHYL)- | 7366 |  | 0.52 | monocyclic aromatics |
| 283 | Benzene, 1-(1,1-dimethylethyl)-3-methyl- | 33711 |  | 0.29 | monocyclic aromatics |
| 284 | BENZENE, 1-(1,5-DIMETHYL-4-HEXENYL)-4-METHYL- | 92139 |  | 1.33 | Alkenes |
| 285 | Benzene, 1-(dimethoxymethyl)-4-(1-methoxy-1-methylethyl)- | 14121776 |  | 12.51 | monocyclic aromatics |
| 286 | Benzene, 1,2,3-trimethoxy-5-(2-propenyl)- | 10248 | C10451 | 6.86 | Alkenes |
| 287 | Benzene, 1-methoxy-3-methyl- | 7530 |  | 0.22 | monocyclic aromatics |
| 288 | Benzene, 1-methyl-4-(1-methylethenyl)- | 62385 |  | 0.39 | Alkenes |
| 289 | BENZENE, METHOXY- | 7519 | C01403 | 0.3 | monocyclic aromatics |
| 290 | Benzene, methyl- | 1140 | C01455 | 0 | monocyclic aromatics |
| 291 | Benzene, methyl(1-methylethyl)- | 10703 |  | 0.19 | monocyclic aromatics |
| 292 | Benzene, tert-butyl- | 7366 |  | 0.13 | monocyclic aromatics |
| 293 | Benzoic acid, 2-hydroxy-, methyl ester | 4133 | C12305 | 3.55 | Esters |
| 294 | Benzoic acid, ethyl ester | 7165 | C01839 | 3.43 | Esters |
| 295 | BENZOIC ACID, METHYL ESTER | 7150 | C20645 | 0.03 | Esters |
| 296 | Bicyclo[1.1.0]butane-1-carboxylic acid, 2,2,4,4-tetramethyl-, methyl ester | 552665 |  | 0.15 | Alkanes |
| 297 | Bicyclo[2.1.0]pentane, 1,4-dimethyl- | 557086 |  | 0.08 | Alkanes |
| 298 | Bicyclo[2.2.1]hept-2-ene, 2,3-dimethyl- | 10720 |  | 0.41 | Alkenes |
| 299 | Bicyclo[2.2.1]heptane, 2-methyl-3-methylene-2-(4-methyl-3-pentenyl)-, (1S-endo)- | 10534 | C09718 | 0.54 | Alkenes |
| 300 | Bicyclo[3.1.1]hept-2-ene, 2,6-dimethyl-6-(4-methyl-3-pentenyl)- | 86608 |  | 51.85 | Alkenes |
| 301 | Bicyclo[3.1.1]hept-2-ene-2-carboxaldehyde, 6,6-dimethyl- | 61130 | C11939 | 2.11 | Alkenes |
| 302 | Bicyclo[4.1.0]heptan-3-ol, 4,7,7-trimethyl-, (1.alpha.,3.alpha.,4.beta.,6.alpha.)- |  |  | 0.12 | Alkanes |
| 303 | Bicyclo[5.2.0]nonane, 4,8,8-trimethyl-2-methylene- | 564732 |  | 0.07 | Alkanes |
| 304 | Bis-(3,5,5-trimethylhexyl) phthalate | 34277 |  | 1.35 | Esters |
| 305 | Butane, 1-cyclopropylidene-5-(tetrahydro-2H-pyran-2-yloxy)- | 558915 |  | 0.03 | Alkanes |
| 306 | Butane, 2,3-dimethoxy-2-methyl- | 552035 |  | 0.08 | Alkanes |
| 307 | BUTANE, 2-METHOXY-3-METHYL- | 547676 |  | 0.1 | Alkanes |
| 308 | Butanedioic acid, compd. with N,N-dimethyl-2-[1-phenyl-1-(2-pyridinyl)ethoxy]ethanamine (1:1) | 11224 |  | 0.11 | Acids |
| 309 | Butanoic acid, 2-methyl- | 8314 | C18319 | 0.17 | Acids |
| 310 | Butanoic acid, 2-methyl-, ethyl ester | 24020 |  | 0.65 | Esters |
| 311 | Butanoic acid, 2-pentenyl ester, (Z)- | 5366233 |  | 1.24 | Alkenes |
| 312 | Butanoic acid, 3-methyl- | 10430 | C08262 | 0.11 | Acids |
| 313 | BUTANOIC ACID, 3-METHYL-, ETHYL ESTER | 7945 | C12290 | 0.45 | Esters |
| 314 | Butanoic acid, 3-methylbut-2-enyl ester | 530923 |  | 0.27 | Alkenes |
| 315 | Butanoic acid, 3-methylbutyl ester | 7795 |  | 0.83 | Esters |
| 316 | BUTANOIC ACID, ETHYL ESTER | 7762 |  | 0.84 | Esters |
| 317 | BUTANOIC ACID, HEXYL ESTER | 17525 |  | 0.23 | Esters |
| 318 | Butanoic acid, phenylmethyl ester | 7650 |  | 0.12 | Esters |
| 319 | Butanoic acid,4-hexen-1-yl ester | 5365695 |  | 0.47 | Alkenes |
| 320 | Butyric acid, 2-phenyl-, 3-methylbut-2-en-1-yl ester | 91699557 |  | 0.24 | Alkenes |
| 321 | CALACORENE | 14038842 |  | 0.11 | Alkenes |
| 322 | Camphene | 6616 | C06076 | 0.21 | Alkenes |
| 323 | Camphor | 2537 | C00809 | 0.8 | Terpenoids |
| 324 | Cedrene | 2537 | C09630 | 0.29 | Terpenoids |
| 325 | Cembrene | 6430770 | C11893 | 0.59 | Alkenes |
| 326 | CIS 3 HEXENYL BUTYRATE | 5352438 |  | 14.56 | Alkenes |
| 327 | CIS 3 HEXENYL LACTATE | 5364231 |  | 0.77 | Alkenes |
| 328 | CIS-.ALPHA.-BISABOLENE | 5352653 |  | 0.18 | Terpenoids |
| 329 | CIS-3-HEXENYL .ALPHA. METHYLBUTYRATE | 5365069 |  | 7.06 | Alkenes |
| 330 | CIS-3-HEXENYL ISOBUTYRATE | 5352539 |  | 3.93 | Alkenes |
| 331 | cis-3-Hexenyl valerate | 5367682 |  | 0.44 | Alkenes |
| 332 | cis-3-methylcyclohexanol | 21599 |  | 0.2 | Alcohols |
| 333 | cis-4-Decenal | 5362620 |  | 0.16 | Alkenals |
| 334 | cis-6-Nonenol | 5362792 |  | 0.35 | Alkenes |
| 335 | cis-7-Tetradecen-1-ol | 5362795 |  | 1.05 | Alkenes |
| 336 | cis-muurola-3,5-diene | 51351708 | C19753 | 0.45 | Alkenes |
| 337 | cis-p-mentha-1(7),8-dien-2-ol | 6429040 |  | 0.11 | Esters |
| 338 | Citronellol epoxide (R or S) | 98467 |  | 0.16 | Alcohols |
| 339 | Cyclobut-1-enylmethanol | 543996 |  | 0.01 | Alkenes |
| 340 | Cyclobutanecarboxylic acid, octyl ester | 544246 |  | 0.21 | Alkanes |
| 341 | Cycloheptanone, 2-methyl- | 543503 |  | 2.89 | Ketones |
| 342 | Cyclohexane, (1,2-dimethylbutyl)- | 521955 |  | 0.03 | Alkanes |
| 343 | Cyclohexane, cyclopropylidene- | 549011 |  | 0.19 | Alkanes |
| 344 | Cyclohexanol | 7966 | C00854 | 3.67 | Alcohols |
| 345 | Cyclohexanol, 1-methyl-4-(1-methylethyl)- | 89437 |  | 0.42 | Alcohols |
| 346 | Cyclohexanol, 3-methyl- | 11566 |  | 0.08 | Alcohols |
| 347 | Cyclohexene, 1-formyl-2-phenylsulfinylmethyl-3,3-dimethyl- | 591955 |  | 0.12 | Alkenes |
| 348 | CYCLOHEXENE-3,6-D2 |  |  | 0.14 | Alkenes |
| 349 | Cyclohexyl hexanoate | 80388 |  | 0.13 | Alcohols |
| 350 | Cyclooctanemethanol | 77196 |  | 0.13 | Alcohols |
| 351 | CYCLOPENTANE, 1-METHYL-3-(1-METHYLETHYL)- | 521465 |  | 0.02 | Alkanes |
| 352 | Cyclopentanemethanol | 77195 |  | 0.06 | Alcohols |
| 353 | Cyclopentanol, 3-methyl- | 86785 |  | 0.17 | Alcohols |
| 354 | CYCLOPROPANE, 1-METHYL-2-(3-METHYLPENTYL)- | 524648 |  | 0.35 | Alkanes |
| 355 | CYCLOPROPANE, PROPYL- | 17014 |  | 0.45 | Alkanes |
| 356 | Decanal | 8175 | C12307 | 1.33 | Aldehydes |
| 357 | DECANE, 2,3,5,8-TETRAMETHYL- | 545611 |  | 0.47 | Alkanes |
| 358 | DECANE, 2-METHYL- | 23415 |  | 2.03 | Alkanes |
| 359 | Decane, 3,7-dimethyl- | 28468 |  | 0.15 | Alkanes |
| 360 | DEHYDROAROMADENDRENE | 91746711 |  | 0.05 | Alkanes |
| 361 | Diazene, bis(1,1-dimethylethyl)- | 70227 |  | 0.03 | polycyclic arenes |
| 362 | DIHYDRO LINALOOL | 6437979 |  | 1.67 | Alcohols |
| 363 | diisobutyl benzene-1,2-dicarboxylate | 6782 |  | 0.26 | Esters |
| 364 | Dimethyl sulfide | 1068 | C00580 | 1.91 | Sulfurs |
| 365 | Dimethylsulfoxonium formylmethylide | 548483 |  | 0.42 | Sulfurs |
| 366 | Dodecanal | 8194 | C02278 | 0.66 | Aldehydes |
| 367 | Dodecane, 1-chloro- | 8192 |  | 0.11 | Alkanes |
| 368 | Dodecane, 2,6,10-trimethyl- | 19773 |  | 0.68 | Alkanes |
| 369 | Dodecane, 2,6,11-trimethyl- | 35768 |  | 0.77 | Alkanes |
| 370 | DODECANE, 4,6-DIMETHYL- | 545627 |  | 0.2 | Alkanes |
| 371 | E-14-Hexadecenal | 5363106 |  | 0.11 | Alkenals |
| 372 | E2-DODECENYLACETATE |  |  | 0.48 | Alkenes |
| 373 | edulan IV |  |  | 0.21 | Alcohols |
| 374 | Eicosane, 2-methyl- | 519146 |  | 0.31 | Alkanes |
| 375 | endo-Borneol | 6552009 | C01411 | 7.52 | Alcohols |
| 376 | Ethane, 1,1'-oxybis- | 3283 | C13240 | 0.05 | Alkanes |
| 377 | Ethanone, 1-(2-methylphenyl)- | 11340 |  | 10.89 | Ketones |
| 378 | Ethanone, 1-(3-methylphenyl)- | 11455 |  | 0.29 | Ketones |
| 379 | Ethanone, 1-(6,10,10-trimethylspiro[4.5]deca-6,8-dien-2-yl)-, (2R-trans)- |  |  | 0.06 | Ketones |
| 380 | Ether, dodecyl isopropyl | 537042 |  | 0.79 | Alkanes |
| 381 | ethyl 2-acetyl-6-cyclopropylidenehexanoate | 132196329 |  | 0.09 | Esters |
| 382 | Ethyl tiglate | 5281163 | C08487 | 0.33 | Esters |
| 383 | Farnesol | 445070 | C06081 | 0.25 | Alcohols |
| 384 | Formamide, N-methyl- | 31254 | C11489 | 0.07 | Others |
| 385 | Furan, 2-methyl- | 10797 |  | 0.54 | Others |
| 386 | Furan, 2-pentyl- | 19602 |  | 15.38 | Others |
| 387 | Germacrene B | 5281519 | C09672 | 0.17 | Alkenes |
| 388 | Germacrene D | 5317570 | C16142 | 0.83 | Alkenes |
| 389 | GERMACRENE-D | 91723653 | C16142 | 7.35 | Alkenes |
| 390 | Globulol | 12304985 |  | 0.24 | Alkenes |
| 391 | Hentriacontane | 12410 | C08376 | 0.36 | Alkanes |
| 392 | Heptadec-8-ene | 520230 |  | 0.43 | Alkenes |
| 393 | HEPTADECANE | 12398 | C01816 | 0.71 | Alkanes |
| 394 | Heptadecane, 2,6,10,15-tetramethyl- | 41209 |  | 1.79 | Alkanes |
| 395 | Heptanal | 8130 | C14390 | 0.13 | Alcohols |
| 396 | HEPTANE | 8900 |  | 0.06 | Alkanes |
| 397 | Heptane, 2,4-dimethyl- | 16656 |  | 0.21 | Alkanes |
| 398 | Heptanediamide, N,N'-di-benzoyloxy- | 569848 |  | 0.15 | Esters |
| 399 | Heptanoic acid, 2-ethyl- | 18648 |  | 0.08 | Acids |
| 400 | Heptanoic acid, 3-hexenyl ester, (Z)- | 5367677 |  | 0.21 | Alkenes |
| 401 | Hexadecane | 11006 | C14499 | 1.14 | Alkanes |
| 402 | Hexadecane, 1,16-dichloro- | 544143 |  | 0.17 | Alkanes |
| 403 | HEXADECANE, 2,6,10,14-TETRAMETHYL- | 12523 |  | 2.45 | Alkanes |
| 404 | Hexadecane, 2-methyl- | 15266 |  | 0.07 | Alkanes |
| 405 | Hexadecanoic acid, ethyl ester | 12366 |  | 0.51 | Alkanes |
| 406 | Hexadecanoic acid, methyl ester | 8181 | C16995 | 0.23 | Alkanes |
| 407 | Hexanal | 6184 | C02373 | 0.58 | Aldehydes |
| 408 | Hexane, 2,3-dimethyl- | 11447 |  | 0.05 | Alkanes |
| 409 | Hexane, 3,3-dimethyl- | 11233 |  | 0.22 | Alkanes |
| 410 | Hexanediamide, N,N'-di-benzoyloxy- | 569844 |  | 0.57 | Esters |
| 411 | Hexanoic acid, 3-hexenyl ester, (Z)- | 5352543 |  | 2.62 | Alkenes |
| 412 | Hexanoic acid, ethyl ester | 31265 |  | 8.01 | Esters |
| 413 | Hydrazine, 1,2-dimethyl- | 1322 |  | 0.2 | Others |
| 414 | Hydrazinecarbodithioic acid, methyl ester | 3034069 |  | 0.92 | Esters |
| 415 | IRON TRITROPOLONATE |  |  | 0.27 | others |
| 416 | ISO BUTYL ALCOHOL | 6560 | C14710 | 0.3 | Alcohols |
| 417 | Isopropyl myristate | 8042 |  | 0.25 | Esters |
| 418 | L-(-)-Menthol | 16666 | C00400 | 0.53 | Alcohols |
| 419 | labda-8(17),13Z-dien-15-ol |  |  | 0.42 | Alcohols |
| 420 | Lanceol, cis | 5352901 |  | 0.18 | Alkenes |
| 421 | LIMONENE DIOXIDE 1 | 232703 |  | 0.37 | Alkenes |
| 422 | l-Limonene | 439250 | C06099 | 7.68 | Alkenes |
| 423 | L-LINALOOL | 443158 | C11388 | 12.1 | Alcohols |
| 424 | menthanol 2 | 21532766 |  | 0.35 | Terpenoids |
| 425 | Methacrylic acid, 2,2,3,3,4,4,4-heptafluorobutyl ester | 83666 |  | 0.33 | Alkenes |
| 426 | Methane, thiobis- | 1068 | C00580 | 0.2 | Alkanes |
| 427 | Methyl 4(E)-Hexenyl Ether |  |  | 0.27 | Alkenes |
| 428 | Methyl peroxybenzoate | 123971982 |  | 0.13 | Esters |
| 429 | Methyl pulegenate | 11830067 |  | 0.04 | Esters |
| 430 | METHYL-7,8-DIDEUTERO-7-NONENOATE |  |  | 0.11 | Alkenes |
| 431 | Myrtenol | 10582 | C11938 | 0.5 | Terpenoids |
| 432 | Naphthalene, 1,7-dimethyl- | 11326 |  | 0.12 | polycyclic arenes |
| 433 | Naphthalene, 1,8-dimethyl- | 11287 | C14703 | 0.21 | polycyclic arenes |
| 434 | Naphthalene, 1-methyl- | 7002 | C14082 | 6.82 | polycyclic arenes |
| 435 | Naphthalene, 2-methyl- | 7055 | C14098 | 0.06 | polycyclic arenes |
| 436 | Naphthalene, decahydro-, cis- | 7044 |  | 0.69 | polycyclic arenes |
| 437 | Naphthalene, decahydro-, trans- | 7044 |  | 1.13 | polycyclic arenes |
| 438 | Naphthalene, trihexyl- | 156701014 |  | 0.1 | polycyclic arenes |
| 439 | n-Dodecylpyridinium chloride | 7717 |  | 0.06 | Alkanes |
| 440 | Neodihydrocarveol | 12072 | C18017 | 0.78 | Alcohols |
| 441 | NERYL ACETATE | 1549025 | C09861 | 0.79 | Esters |
| 442 | NERYL ACETONE | 19256382 |  | 0.41 | Ketones |
| 443 | Nonadecane | 12401 |  | 0.22 | Alkanes |
| 444 | Nonanal | 31289 |  | 8.95 | Aldehydes |
| 445 | Nonane, 2-methyl- | 13379 |  | 0.1 | Alkanes |
| 446 | Nonane, 3,7-dimethyl- | 28458 |  | 0.14 | Alkanes |
| 447 | Nonanoic acid, ethyl ester | 31251 |  | 0.27 | Alkanes |
| 448 | Octadecanoic acid, (2-phenyl-1,3-dioxolan-4-yl)methyl ester | 569173 |  | 0.04 | Alkanes |
| 449 | Octadecanoic acid, 1-[(tetradecyloxy)carbonyl]pentadecyl ester | 622422 |  | 0.08 | Alkanes |
| 450 | Octadecanoic acid, ethenyl ester | 66077 |  | 1.78 | Alkenes |
| 451 | Octadecanoic acid, ethyl ester | 8122 |  | 0.14 | Alkanes |
| 452 | Octanal | 454 | C01545 | 1.45 | Aldehydes |
| 453 | Octanal, 7-methoxy-3,7-dimethyl- | 19223 |  | 0.56 | Aldehydes |
| 454 | Octane | 356 | C01387 | 0.52 | Alkanes |
| 455 | Octane, 1-(1-ethoxyethoxy)- | 552095 |  | 0.14 | Alkanes |
| 456 | OCTANE, 2,7-DIMETHYL- | 14070 | C11908 | 0.16 | Alkanes |
| 457 | OCTANE, 3,3-DIMETHYL- | 138117 |  | 0.31 | Alkanes |
| 458 | Octane, 3,4,5,6-tetramethyl- | 526427 |  | 0.16 | Alkanes |
| 459 | Octane, 3,5-dimethyl- | 139989 |  | 0.9 | Alkanes |
| 460 | Octane, 3-ethyl-2,7-dimethyl- | 537329 |  | 0.19 | Alkanes |
| 461 | Octane, 6-ethyl-2-methyl- | 537768 |  | 0.21 | Alkanes |
| 462 | o-Cymene | 10703 |  | 1.12 | Quinone |
| 463 | o-Mentha-1(7),8-dien-3-ol | 564552 |  | 0.05 | Terpenoids |
| 464 | OXACYCLOHEXADECANE-2,13-DIONE 13-OXIME | 99859 |  | 0.64 | Ketones |
| 465 | Oxacyclotetradeca-4,11-diyne | 560917 |  | 0.14 | Alkanes |
| 466 | Oxalic acid, cyclohexylmethyl decyl ester | 6421721 |  | 0.07 | Esters |
| 467 | Oxetane, 2-ethyl-3-methyl- | 40894 |  | 0.12 | Alkanes |
| 468 | Oxime-, methoxy-phenyl- | 9602988 |  | 80.4 | Others |
| 469 | Oxirane, 2,2'-(1,4-butanediyl)bis- | 17048 |  | 0.1 | Ketones |
| 470 | PENTADECANE | 12391 | C08388 | 1.34 | Alkanes |
| 471 | PENTADECANE, 2,6,10,14-TETRAMETHYL- | 15979 |  | 1.4 | Alkanes |
| 472 | PENTAMETHYLENE SULFOXIDE-D4 | 534965 |  | 0.13 | Sulfurs |
| 473 | Pentanal, 5-(benzoyloxy)- | 569875 |  | 0.19 | Aldehydes |
| 474 | PENTANE, 3-METHYL- | 7282 |  | 0.06 | Alkanes |
| 475 | Pentanoic acid, ethyl ester | 10882 |  | 0.13 | Esters |
| 476 | Pentatriacontane | 12413 |  | 0.65 | Alkanes |
| 477 | Peracetic Acid | 6585 | D03467 | 0.37 | Acids |
| 478 | Perfluoropropionic acid, TMS derivative | 12452932 |  | 0.09 | Acids |
| 479 | Petasitene | 636697 |  | 0.9 | Alkenes |
| 480 | Phenol | 996 | C15584 | 0.99 | Phenols |
| 481 | Phenol, (1,1-dimethylethyl)-4-methoxy- | 8456 |  | 0.06 | Phenols |
| 482 | Phenol, 2,6-bis(1,1-dimethylethyl)-4-ethyl- | 20087 |  | 0.26 | Phenols |
| 483 | Phenol, 2,6-bis(1,1-dimethylethyl)-4-methyl- | 31404 | C14693 | 1.08 | Phenols |
| 484 | Phenol, 2-methoxy- | 460 | C01502 | 4.07 | Phenols |
| 485 | Phenol, 3,5-bis(1,1-dimethylethyl)- | 70825 |  | 0.12 | Phenols |
| 486 | PHENYL ACETALDEHYDE | 998 | C00601 | 0.31 | Aldehydes |
| 487 | Phosphine oxide, triphenyl- | 13097 |  | 1.2 | polycyclic arenes |
| 488 | Phosphinothioic anidee, di(tert-butyl)-N-isopropyl- | 546346 |  | 168.24 | Acids |
| 489 | Pivalic acid, 6-chlorohexyl ester | 11172065 |  | 0.17 | Esters |
| 490 | PROPANE, 2-METHOXY-2-METHYL- | 15413 | C11344 | 0.06 | Alkanes |
| 491 | Propanoic acid, 2-methyl-, 2-methylbutyl ester | 97883 |  | 0.18 | Esters |
| 492 | Propen-2-yl nonaflate |  |  | 0.12 | Alkenes |
| 493 | Pyrazine, 2-methoxy-3-(1-methylethyl)- | 33166 |  | 1.02 | Nucleotides and derivatives |
| 494 | Pyridinium, 1-hexadecyl-, chloride, monohydrate | 22324 | C11307 | 0.15 | Alkanes |
| 495 | Rose oxide | 27866 |  | 0.61 | others |
| 496 | Santolina triene | 519872 |  | 0.04 | Alkenes |
| 497 | Spiro[4.5]dec-7-ene, 1,8-dimethyl-4-(1-methylethenyl)-, [1S-(1.alpha.,4.beta.,5.alpha.)]- | 520091 |  | 0.38 | Alkenes |
| 498 | SPIRO[5.5]UNDECA-1,8-DIENE, 1,5,5,9-TETRAMETHYL-, (R)- | 519725 |  | 1.21 | Alkenes |
| 499 | Spiro[androst-5-ene-17,1'-cyclobutan]-2'-one, 3-hydroxy-, (3.beta.,17.beta.)- | 534435 |  | 0.42 | others |
| 500 | Spiropentane, butyl- | 22566 |  | 0.05 | Alkanes |
| 501 | Styrene | 7501 | C19506 | 0.25 | Alkenes |
| 502 | Succinic acid, butyl trans-hex-3-enyl ester | 91697924 |  | 0.22 | Alkenes |
| 503 | Terpineol | 17100 | C16772 | 1.64 | Alcohols |
| 504 | Tetracosane, 3-ethyl- | 294709 |  | 0.15 | Alkanes |
| 505 | Tetracosanoic acid, methyl ester | 75546 |  | 0.41 | Esters |
| 506 | Tetracyclo[5.2.1.0(2,6).0(3,5)]decane, 4,4-dimethyl- | 565066 |  | 0.13 | Alkanes |
| 507 | Tetradecanal | 31291 |  | 0.94 | Aldehydes |
| 508 | Tetradecane | 12389 |  | 1.82 | Alkanes |
| 509 | Tetradecanoic acid, 12-methyl-, methyl ester | 21206 |  | 0.21 | Alkanes |
| 510 | Torreyol | 3084311 |  | 0.05 | Phenols |
| 511 | trans,Trans-nona-2,4-dienol | 5364586 |  | 1.75 | Alkenes |
| 512 | trans-1,2-Di(1-methylethyl)cyclopropane |  |  | 0.06 | Alkanes |
| 513 | trans-2-cis-6-Nonadien-1-ol | 5362833 |  | 0.1 | Alcohols |
| 514 | trans-3-Hexen-1-ol, trifluoroacetate | 91694379 |  | 2.45 | Alkenes |
| 515 | trans-Caryophyllene | 5281515 | C09629 | 9.39 | Terpenoids |
| 516 | trans-chrysanthemal | 6432223 |  | 0.08 | Aldehydes |
| 517 | TRANS-ISOELEMICIN | 5318557 | C16975 | 0.22 | others |
| 518 | TRANS-LINALOOL OXIDE | 6432254 |  | 0.28 | Alcohols |
| 519 | trans-Rose oxide | 7093102 |  | 0.15 | Monocyclic aromatics |
| 520 | Tricosane | 12534 | C17433 | 0.28 | Alkanes |
| 521 | Tricyclo[2.2.1.0(2,6)]heptane, 1,7-dimethyl-7-(4-methyl-3-pentenyl)- | 6431166 |  | 1.15 | Terpenoids |
| 522 | tridecanal | 25311 |  | 0.14 | Aldehydes |
| 523 | Tridecane | 12388 | C13834 | 0.09 | Alkanes |
| 524 | Undecanal | 28454 |  | 2.11 | Alkanes |
| 525 | Undecane, 2,8-dimethyl- | 519384 |  | 0.17 | Alkanes |
| 526 | UNDECANE, 4,8-DIMETHYL- | 28454 |  | 0.05 | Alkanes |
| 527 | Undecanone | 8163 | C01875 | 0.93 | Ketones |
| 528 | UREA | 1176 | C00086 | 2.33 | Others |
| 529 | Valeric acid, 3-methylbut-2-enyl ester | 530629 |  | 0.19 | Alkenes |
| 530 | Z-2-Dodecenol | 5364955 |  | 0.13 | Alkenes |
| 531 | Z-3-hexenyl tiglate | 5352469 |  | 4.75 | Alkenes |
| 532 | Zingiberene | 92776 | C09750 | 0.22 | Alkenes |
