# Supplementary material for: Comparative analysis of flavonoids, polyphenols and volatiles in roots, stems and leaves of five mangroves
Source: PeerJ. 2023 Jun 22;11:e15529. doi: 10.7717/peerj.15529 (PMC10290835; doi:10.7717/peerj.15529)
Supplement: Supplemental Information 8 [file peerj-11-15529-s008.docx]

| **Species_parts** | **Classes** | **Total content**/% |
| --- | --- | --- |
| AI_leaf | Alcohols | 6.12 |
| AI_leaf | Aldehydes | 0.84 |
| AI_leaf | Alkanes | 4.76 |
| AI_leaf | Alkenes | 19.13 |
| AI_leaf | Esters | 2.51 |
| AI_leaf | Ketones | 4.74 |
| AI_leaf | Monocyclic aromatics | 0.3 |
| AI_leaf | Nucleotides and derivatives | 0.49 |
| AI_leaf | Others | 4.86 |
| AI_leaf | Phenols | 0.26 |
| AI_leaf | polycyclic arenes | 0.19 |
| AI_leaf | Terpenoids | 0.32 |
| AI_root | Acids | 0.15 |
| AI_root | Alcohols | 3.71 |
| AI_root | Aldehydes | 1.81 |
| AI_root | Alkanes | 2.85 |
| AI_root | Alkenes | 3.49 |
| AI_root | Aromatic hydrocarbons | 0.54 |
| AI_root | Esters | 4.71 |
| AI_root | Ketones | 3.53 |
| AI_root | Monocyclic aromatics | 1.87 |
| AI_root | Nucleotides and derivatives | 1.02 |
| AI_root | Others | 15.75 |
| AI_root | polycyclic arenes | 1.2 |
| AI_root | Sulfurs | 0.42 |
| AI_root | Terpenoids | 0.78 |
| AI_stem | Alcohols | 4.87 |
| AI_stem | Aldehydes | 1.96 |
| AI_stem | Alkanes | 3.55 |
| AI_stem | Alkenes | 2.98 |
| AI_stem | Aromatic hydrocarbons | 0.73 |
| AI_stem | Esters | 3 |
| AI_stem | Ketones | 5.11 |
| AI_stem | Monocyclic aromatics | 1.92 |
| AI_stem | Others | 20.58 |
| AI_stem | polycyclic arenes | 0.83 |
| AI_stem | Terpenoids | 0.61 |
| BG_leaf | Acids | 16.54 |
| BG_leaf | Alcohols | 9.96 |
| BG_leaf | Aldehydes | 0.08 |
| BG_leaf | Alkanes | 0.34 |
| BG_leaf | Alkenals | 0.11 |
| BG_leaf | Alkenes | 41.64 |
| BG_leaf | Aromatic hydrocarbons | 0.05 |
| BG_leaf | Esters | 19.2 |
| BG_leaf | Ketones | 2.23 |
| BG_leaf | Monocyclic aromatics | 0.25 |
| BG_leaf | Others | 2.09 |
| BG_leaf | Terpenoids | 0.17 |
| BG_root | Acids | 44.75 |
| BG_root | Alcohols | 7.6 |
| BG_root | Aldehydes | 0.84 |
| BG_root | Alkanes | 1.42 |
| BG_root | Alkenals | 0.22 |
| BG_root | Alkenes | 18.41 |
| BG_root | Aromatic hydrocarbons | 0.11 |
| BG_root | Esters | 0.36 |
| BG_root | Ketones | 3.77 |
| BG_root | Monocyclic aromatics | 0.54 |
| BG_root | Others | 5.85 |
| BG_root | Phenols | 0.2 |
| BG_root | polycyclic arenes | 0.28 |
| BG_root | Terpenoids | 1.17 |
| BG_stem | Acids | 54.75 |
| BG_stem | Alcohols | 7.53 |
| BG_stem | Aldehydes | 0.24 |
| BG_stem | Alkanes | 0.31 |
| BG_stem | Alkenes | 11.27 |
| BG_stem | Esters | 0.34 |
| BG_stem | Ketones | 6.79 |
| BG_stem | Monocyclic aromatics | 0.38 |
| BG_stem | Others | 5.07 |
| BG_stem | polycyclic arenes | 0.25 |
| BG_stem | Terpenoids | 0.61 |
| AC_leaf | Acids | 0.8 |
| AC_leaf | Alcohols | 3.58 |
| AC_leaf | Aldehydes | 1.11 |
| AC_leaf | Alkanes | 2.09 |
| AC_leaf | Alkenes | 36.66 |
| AC_leaf | Aromatic hydrocarbons | 0.67 |
| AC_leaf | Esters | 18.98 |
| AC_leaf | Ketones | 0.21 |
| AC_leaf | Monocyclic aromatics | 1.51 |
| AC_leaf | Others | 5.45 |
| AC_leaf | Phenols | 0.13 |
| AC_leaf | polycyclic arenes | 2.15 |
| AC_leaf | Sulfurs | 2.78 |
| AC_leaf | Terpenoids | 0.12 |
| AC_root | Acids | 0.11 |
| AC_root | Alcohols | 15.91 |
| AC_root | Aldehydes | 5.22 |
| AC_root | Alkanes | 5.63 |
| AC_root | Alkenals | 1.86 |
| AC_root | Alkenes | 28.78 |
| AC_root | Aromatic hydrocarbons | 0.46 |
| AC_root | Esters | 0.57 |
| AC_root | Ketones | 2.13 |
| AC_root | Monocyclic aromatics | 1.19 |
| AC_root | Others | 2.54 |
| AC_root | Phenols | 0.65 |
| AC_root | polycyclic arenes | 1.43 |
| AC_root | Terpenoids | 1.68 |
| AC_stem | Alcohols | 2.22 |
| AC_stem | Aldehydes | 0.69 |
| AC_stem | Alkanes | 2.48 |
| AC_stem | Alkenals | 0.03 |
| AC_stem | Alkenes | 74.34 |
| AC_stem | Aromatic hydrocarbons | 0.83 |
| AC_stem | Esters | 0.91 |
| AC_stem | Ketones | 3.97 |
| AC_stem | Monocyclic aromatics | 0.54 |
| AC_stem | Nucleotides and derivatives | 0.06 |
| AC_stem | Others | 1.78 |
| AC_stem | polycyclic arenes | 1 |
| AC_stem | Sulfurs | 0.38 |
| AC_stem | Terpenoids | 1.17 |
| KC_leaf | Alcohols | 10.04 |
| KC_leaf | Aldehydes | 1.58 |
| KC_leaf | Alkanes | 1.43 |
| KC_leaf | Alkenes | 49.28 |
| KC_leaf | Esters | 2.33 |
| KC_leaf | Ketones | 0.12 |
| KC_leaf | Monocyclic aromatics | 1.04 |
| KC_leaf | Others | 8.49 |
| KC_leaf | polycyclic arenes | 0.61 |
| KC_leaf | Sulfurs | 0.13 |
| KC_leaf | Terpenoids | 0.79 |
| KC_root | Acids | 29.6 |
| KC_root | Alcohols | 24.43 |
| KC_root | Aldehydes | 1.33 |
| KC_root | Alkanes | 2.25 |
| KC_root | Alkenals | 0.83 |
| KC_root | Alkenes | 5.96 |
| KC_root | Aromatic hydrocarbons | 0.35 |
| KC_root | Esters | 1.36 |
| KC_root | Esters | 0.57 |
| KC_root | Ketones | 0.47 |
| KC_root | Monocyclic aromatics | 0.68 |
| KC_root | Others | 7.93 |
| KC_root | Phenols | 5.26 |
| KC_root | polycyclic arenes | 0.77 |
| KC_root | Terpenoids | 1.4 |
| KC_stem | Acids | 22.88 |
| KC_stem | Alcohols | 6.75 |
| KC_stem | Aldehydes | 1.28 |
| KC_stem | Alkanes | 2.21 |
| KC_stem | Alkenals | 0.13 |
| KC_stem | Alkenes | 13.2 |
| KC_stem | Aromatic hydrocarbons | 0.59 |
| KC_stem | Esters | 3.17 |
| KC_stem | Ketones | 2.87 |
| KC_stem | Monocyclic aromatics | 1.1 |
| KC_stem | Others | 6.16 |
| KC_stem | Phenols | 0.13 |
| KC_stem | polycyclic arenes | 0.82 |
| KC_stem | Quinone | 1.12 |
| KC_stem | Terpenoids | 6.85 |
| AM_leaf | Acids | 0.09 |
| AM_leaf | Alcohols | 6.93 |
| AM_leaf | Aldehydes | 0.69 |
| AM_leaf | Alkanes | 2.25 |
| AM_leaf | Alkenes | 32.71 |
| AM_leaf | Esters | 1.02 |
| AM_leaf | Esters | 0.15 |
| AM_leaf | Ketones | 13.91 |
| AM_leaf | Monocyclic aromatics | 1.97 |
| AM_leaf | Others | 2.25 |
| AM_leaf | polycyclic arenes | 2.76 |
| AM_leaf | Sulfurs | 0.71 |
| AM_leaf | Terpenoids | 3.25 |
| AM_root | Acids | 0.24 |
| AM_root | Alcohols | 15.21 |
| AM_root | Aldehydes | 1.84 |
| AM_root | Alkaloids | 0.21 |
| AM_root | Alkanes | 4.44 |
| AM_root | Alkenals | 1.15 |
| AM_root | Alkenes | 22.72 |
| AM_root | Aromatic hydrocarbons | 0.27 |
| AM_root | Esters | 3.78 |
| AM_root | Ketones | 3.75 |
| AM_root | Monocyclic aromatics | 1.12 |
| AM_root | Others | 10.1 |
| AM_root | Phenols | 0.08 |
| AM_root | polycyclic arenes | 0.25 |
| AM_root | Sulfurs | 0.33 |
| AM_root | Terpenoids | 2.76 |
| AM_stem | Acids | 0.08 |
| AM_stem | Alcohols | 11.19 |
| AM_stem | Aldehydes | 1.45 |
| AM_stem | Alkanes | 3.3 |
| AM_stem | Alkenes | 27.7 |
| AM_stem | Aromatic hydrocarbons | 1.14 |
| AM_stem | Esters | 0.17 |
| AM_stem | Ketones | 10.45 |
| AM_stem | Monocyclic aromatics | 0.11 |
| AM_stem | Others | 4.76 |
| AM_stem | Polycyclic arenes | 0.16 |
| AM_stem | Terpenoids | 2.24 |
