# Supplementary material for: Comparative analysis of flavonoids, polyphenols and volatiles in roots, stems and leaves of five mangroves
Source: PeerJ. 2023 Jun 22;11:e15529. doi: 10.7717/peerj.15529 (PMC10290835; doi:10.7717/peerj.15529)
Supplement: Supplemental Information 9 [file peerj-11-15529-s009.docx]

| **Source** | **Sum of Squares** | **df** | **Mean square** | **F Value** | **p-value Prob > F** |
| --- | --- | --- | --- | --- | --- |
| Model | 11374.24 | 14 | 812.4459 | 7568899 | < 0.0001 |
| A(species) | 10147.18 | 4 | 2536.795 | 23633262 | < 0.0001 |
| B(parts) | 41.99617 | 2 | 20.99809 | 195622.1 | < 0.0001 |
| A×B | 1185.065 | 8 | 148.1332 | 1380036 | < 0.0001 |
| Residual | 0.003006 | 28 | 0.000107 |  |  |
