# Supplementary material for: Comparative analysis of flavonoids, polyphenols and volatiles in roots, stems and leaves of five mangroves
Source: PeerJ. 2023 Jun 22;11:e15529. doi: 10.7717/peerj.15529 (PMC10290835; doi:10.7717/peerj.15529)
Supplement: Supplemental Information 10 [file peerj-11-15529-s010.docx]

| **Source** | **Sum of Squares** | **df** | **Mean square** | **F Value** | **p-value Prob > F** |
| --- | --- | --- | --- | --- | --- |
| Model | 3531.91 | 14 | 252.2793 | 59003.94 | < 0.0001 |
| A(species) | 579.7967 | 4 | 144.9492 | 33901.21 | < 0.0001 |
| B(parts) | 1096.26 | 2 | 548.1301 | 128198.5 | < 0.0001 |
| A×B | 1855.853 | 8 | 231.9816 | 54256.66 | < 0.0001 |
| Residual | 0.119718 | 28 | 0.004276 |  |  |
