# Supplementary material for: Comparative analysis of flavonoids, polyphenols and volatiles in roots, stems and leaves of five mangroves
Source: PeerJ. 2023 Jun 22;11:e15529. doi: 10.7717/peerj.15529 (PMC10290835; doi:10.7717/peerj.15529)
Supplement: Supplemental Information 11 [file peerj-11-15529-s011.docx]

| **No.** | **Compounds** | **HMDB ID** | **PubChem CID** | **KEGG ID** |
| --- | --- | --- | --- | --- |
| 1 | 1-hexanol | 0012971 | 8103 | C00854 |
| 2 | ethyl pentyl ketone | 0031295 | 246728 | C17145 |
| 3 | (+)-alpha-barbatene | - | 135626207 | C19740 |
| 4 | 1,2-dihydroxy-8-methylnaphthalene | - | 854320 | C14084 |
| 5 | (e)-3-hexen-1-ol | 0030003 | 5281167 | C08492 |
| 6 | cedrol | - | 65575 | C09631 |
| 7 | alpha-copaene | 0061851 | 12303902 | C09639 |
| 8 | d-limonene | 0003375 | 439250 | C06099 |
| 9 | (s)-3-octanol | 0030070 | 11527 | C17144 |
| 10 | p-menth-1-en-4-ol | 0035833 | 11230 | C17073 |
| 11 | phytanic acid | 0000801 | 26840 | C01607 |
| 12 | amorpha-4,11-diene | - | 11052747 | C16028 |
| 13 | menthol | 0003352 | 16666 | C00400 |
| 14 | heptadecane | 0059830 | 12398 | C01816 |
| 15 | eucalyptol | 0004472 | 2758 | C09844 |
| 16 | methyl 2-hydroxybenzoate | 0034172 | 4133 | C12305 |
| 17 | 1-methylnaphthalene | 0032860 | 7002 | C14082 |
